# Beyond severe iodinated contrast allergy: a contrast-free endoscopic ultrasound-guided rendezvous approach

**DOI:** 10.1055/a-2616-8277

**Published:** 2025-06-26

**Authors:** Elena De Cristofaro, Jérôme Rivory, Charles-Eric Ber, Florian Rostain, Alexandru Lupu, Mathieu Pioche

**Affiliations:** 1 Gastroenterology Unit, Department of Systems Medicine, University of Rome Tor Vergata, Rome, Italy; 2 Department of Gastroenterology and Endoscopy, Hôpital Edouard Herriot, Hospices Civils de Lyon, Lyon, France; 3 Department of Anesthesiology and Hepatogastroenterology, Edouard Herriot Hospital, Lyon, France

Endoscopic retrograde cholangiopancreatography (ERCP) is a standard technique for managing various pancreatobiliary conditions. When ERCP fails or is not feasible, endoscopic ultrasound-guided (EUS) biliary drainage has emerged as a valuable alternative 1 . Both procedures typically rely on fluoroscopic guidance using iodinated contrast media to visualize the biliary and pancreatic ducts, essential for diagnosis and intervention.

Although rare, allergies to iodinated contrast can be severe. Reactions are mostly mild to moderate, with life-threatening events occurring in only 0.01%–0.02% of cases. In most situations, premedication with steroids and antihistamines is sufficient 2 3 4 .

We report the case of a 45-year-old woman with pancreatic neoplasia and jaundice, referred for biliary drainage. She had a confirmed history of severe anaphylactic shock to iodinated contrast media, with a formal contraindication confirmed by the hospital’s allergy unit ( Video 1 ).

Contrast-free endoscopic ultrasound-guided rendezvous approach.Video 1

Due to the impossibility of performing ERCP, an EUS-guided rendezvous approach was adopted. After identifying the pancreatic lesion and confirming absence of cystic duct invasion, an opaque iron wire was fixed on the skin to mark the optimal biliary access point. Under EUS guidance, the dilated bile duct was punctured at the cystic confluence ( Fig. 1 ), and a guidewire was advanced toward the duodenum and retrieved with forceps using a duodenoscope.

**Fig. 1:**
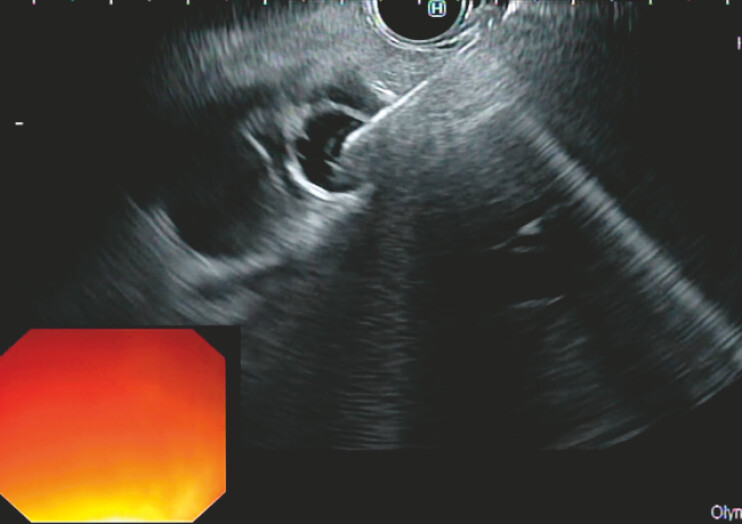
Puncture of the dilated bile duct at the cystic confluence under endoscopic ultrasound guidance.

A second guidewire was placed retrogradely in the bile duct. Positioning of a fully covered metal stent was assisted by the radio-opaque marker of the first scope wire placed in the cystic duct. Good biliary flow was confirmed, with clear visualization of the restricted section of the prothesis within the stenotic area at fluoroscopy ( Fig. 2 ). No adverse events occurred. Biochemical improvement was evident by Day 2.

**Fig. 2:**
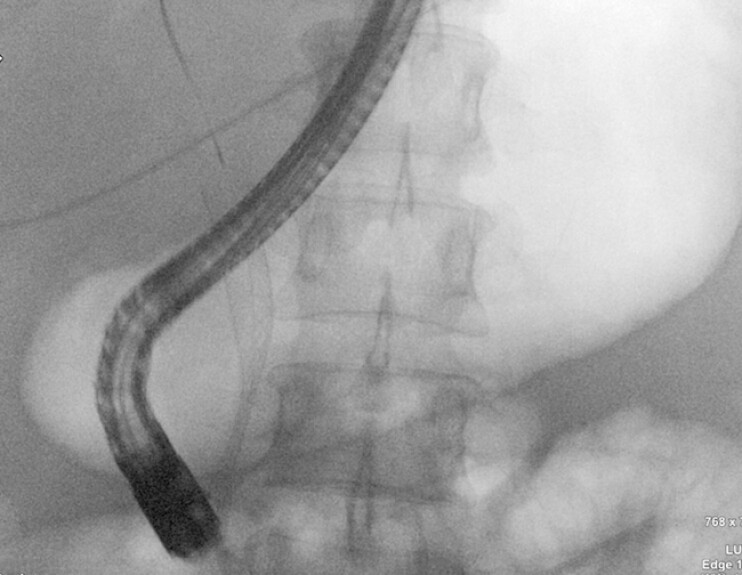
Fluoroscopic visualization of the restricted portion of the prosthesis within the stenotic area.

This case highlights the feasibility and safety of EUS-guided rendezvous as an alternative when ERCP is contraindicated. The procedure enabled successful biliary drainage without iodinated contrast, demonstrating its potential role in managing pancreatobiliary obstructions in high-risk patients.

Endoscopy_UCTN_Code_TTT_1AR_2AK

Endoscopy_UCTN_Code_TTT_1AS_2AH

## Notes

Endoscopy E-Videos https://eref.thieme.de/e-videos
*E-Videos* is an open access online section of the journal *Endoscopy* , reporting on interesting cases and new techniques in gastroenterological endoscopy. All papers include a high-quality video and are published with a Creative Commons CC-BY license. Endoscopy E-Videos qualify for HINARI discounts and waivers and eligibility is automatically checked during the submission process. We grant 100% waivers to articles whose corresponding authors are based in Group A countries and 50% waivers to those who are based in Group B countries as classified by Research4Life (see: https://www.research4life.org/access/eligibility/ ). This section has its own submission website at https://mc.manuscriptcentral.com/e-videos .
